# Genome-Wide Characterization of Somatic Mutation Patterns in Cloned Dogs Reveals Implications for Neuronal Function, Tumorigenesis, and Aging

**DOI:** 10.3390/genes15060801

**Published:** 2024-06-18

**Authors:** Seung-Wan Woo, Miju Kim, Dayeon Kang, Yong-ho Choe, Seong-Ju Oh, Are-Sun You, Sung-Lim Lee, Jaemin Kim

**Affiliations:** 1 Division of Applied Life Science, Gyeongsang National University, Jinju 52828, Republic of Korea; wooswan123@naver.com (S.-W.W.); jimn907@naver.com (D.K.); 2 Institute of Agriculture and Life Sciences, Gyeongsang National University, Jinju 52828, Republic of Korea; kmjxoz@naver.com; 3 College of Veterinary Medicine, Gyeongsang National University, Jinju 52828, Republic of Korea; yhchoe@gnu.ac.kr (Y.-h.C.); osj414@gnu.ac.kr (S.-J.O.); sllee@gnu.ac.kr (S.-L.L.); 4 Division of Animal Diseases & Health, National Institute of Animal Science, RDA, Wanju 55365, Republic of Korea; aresun@korea.kr

**Keywords:** cloned dog, somatic mutation, aging

## Abstract

Studies on somatic mutations in cloned animals have revealed slight genetic variances between clones and their originals, but have yet to identify the precise effects of these differences within the organism. Somatic mutations contribute to aging and are implicated in tumor development and other age-related diseases. Thus, we compared whole genome sequencing data from an original dog with that of cloned dogs, identifying candidate somatic mutations that were disproportionately located within genes previously implicated in aging. The substitutional signature of cloning-specific somatic mutations mirrored the uniform distribution characteristic of the signature associated with human aging. Further analysis of genes revealed significant enrichment of traits associated with body size as well as the molecular mechanisms underlying neuronal function and tumorigenesis. Overall, the somatic mutations found in cloned dogs may indicate a conserved mechanism driving aging across species and a broad spectrum of pathway alterations.

## 1. Introduction

When producing genetically identical individuals, animals are cloned using somatic cell nuclear transfer (SCNT), in which donor somatic cells are transferred to enucleated oocytes [1]. Cloned animals are supposed to be genetically or phenotypically identical to the original animals, but sometimes they exhibit abnormalities not observed in the originals [2,3]. Researchers have attempted to understand the mechanisms of reprogramming by investigating the causes of mutations or abnormalities and the recovery of telomere length in cloned animals compared to the originals [4,5,6]. A study on somatic mutations in cloned animals demonstrated minor genetic differences between clones and their origin animals, but did not determine the specific effects of these differences within the organism [7].

Somatic mutations cause aging [8,9]. Unlike germline mutations, which are inherited from parents, somatic mutations are acquired during an organism’s lifetime. The accumulation can reduce cellular function, leading to issues such as disease [10,11,12]. As aging progresses, the incidence of disease (e.g., cancer and dementia) tends to increase, which has been attributed to the influence of somatic mutations [13,14,15]. The literature has also suggested that factors such as epigenetic reprogramming, telomere length, accumulation of damaged macromolecules, and mutations in nuclear and mitochondrial DNA contribute to the aging of cloned animals [16]. Therefore, we aimed to characterize the genome-wide somatic mutations that exist only in cloned animals, as well as their associated traits, to infer the molecular mechanisms attributed to cloning. Thus, we aimed to determine the somatic mutations unique to cloned dogs that might have resulted in them being different from the original dogs. Despite the two groups sharing concordant overall genotypes, we investigated specific sites in the genotype that differed between the cloned dogs and the original dog.

## 2. Results and Discussion

### 2.1. Cloned Dog Data for Somatic Mutation Filtering

**Figure 1:**
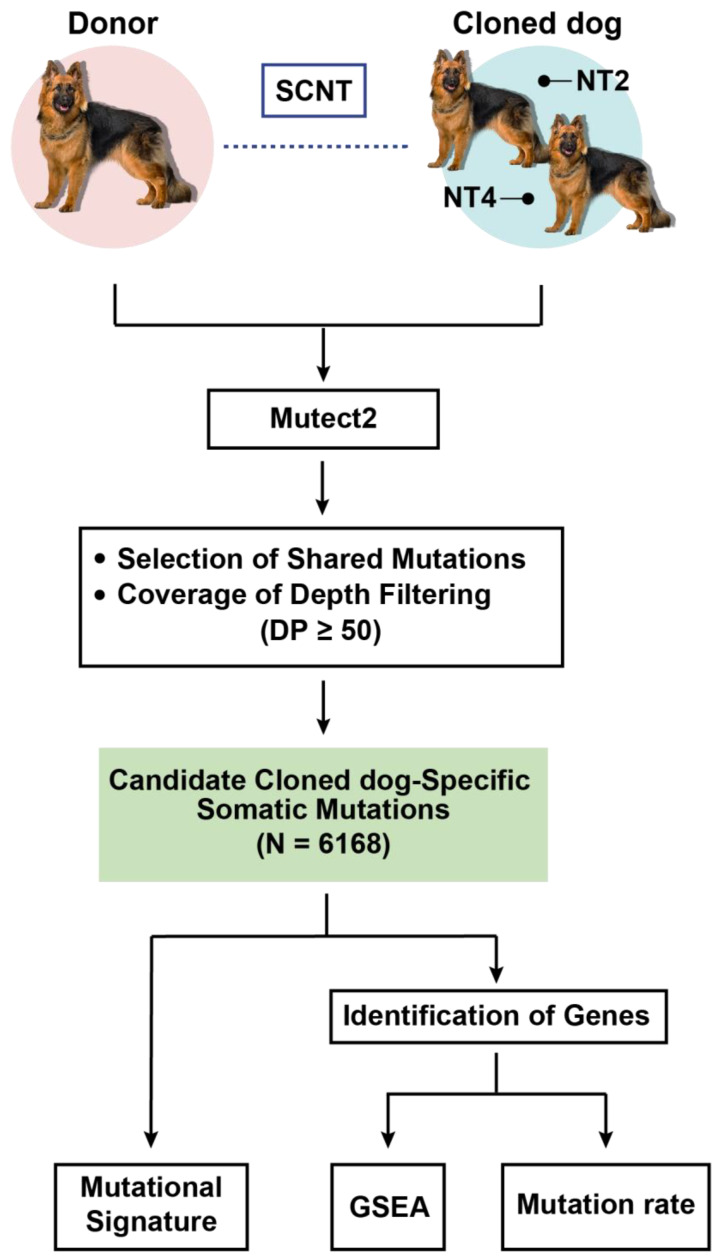
Somatic mutation filtering flow chart. Using SCNT (Somatic Cell Nuclear Transfer), we cloned two healthy cloned dogs, NT2 and NT4, from the original dog (Donor). We identified somatic mutations from the whole blood data of the Donor and the two cloned dogs using Mutect2. Subsequently, variants were filtered based on their DP, and variants present in both cloned dogs—totaling 6168—were classified as candidate somatic mutations. Using these candidate somatic mutations, we examined the mutational signature. Further, among the candidate somatic mutations, we selected those with somatic mutations within genes to undergo the gene set enrichment analysis and mutation rate calculations.

If an individual is cloned, the genetic information of the original and the cloned animals is identical. Therefore, any differences between cloned and original animals are likely due to mutations unique to the cloned animal. We refer to specific variations observed uniquely in cloned dogs, distinct from the donor, as somatic mutations. In our previous study, cloned dogs were generated using Somatic Cell Nuclear Transfer (SCNT) from a donor [17]. Whole genome sequencing data were obtained by using the Illumina sequencing platform to analyze the blood of the donor and its cloned dogs (NT2 and NT4), which were clinically diagnosed as completely normal. The sequenced reads were mapped to the canine reference genome CanFam3.1, achieving an average alignment rate of 98.2%. The sequenced samples exhibited an average depth of coverage of ~33× (Supplementary Table S1). To discern somatic mutations attributable to the cloning process, Mutect2 was used to identify somatic mutations [18]. We compared the genomes of the donor and each cloned dog and identified 42,184 (NT2) and 38,821 (NT4) heterozygous sites specific to each cloned dog. To remove the potential false positives, only variants commonly discovered in both cloned dogs at a high depth of coverage of at least 50 were considered as candidates. As a result, 6168 variants spanning a total of 420 annotated genes remained and were considered candidate somatic mutations (Figure 1). Of these 6168 variants, 38 were predicted to be moderate- or high-effect variants based on the snpEff annotation [19]. We then leveraged publicly available sequences from 196 domestic dogs to estimate the general population allele frequency and found that 13 of 38 showed frequencies of 0 to <0.01 [20]. (Supplementary Table S2).

### 2.2. The Mutational Signature of Cloned Dog-Specific Somatic Mutations

In studies of somatic mutations in tumors, the analysis of mutational signatures has enabled the identification of distinct mutation patterns [21,22]. Although mutational signature analysis is primarily conducted in cancer research, it is also conducted in studies on aging and differences between populations, as it provides insights into the etiology of mutations caused by somatic mutation [23,24,25]. In human tumor research, 67 single-base substitution (SBS) mutational signatures have been extracted, with 49 considered to have a probable biological origin [21].

**Figure 2:**
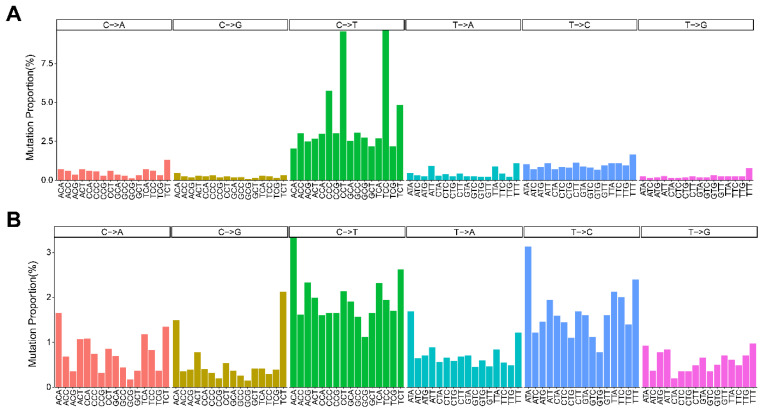
Mutational signature and mutational proportion. The CTVT somatic mutations exhibit a notable transition from C to T in their mutational signature (**A**). In the mutational signature of the candidate somatic mutations, although all mutation patterns are ubiquitous and flat, C-to-T and T-to-C shifts are notable (**B**).

Thus, we examined the substitution signatures of the 6168 identified somatic mutations within their corresponding trinucleotide contexts. The mutation pattern observed in the cloned dogs did not exhibit a pronounced bias for specific base substitutions, mirroring the uniform distribution characteristic of the SBS5 signature observed in humans (Figure 2). Intriguingly, though the precise mutational mechanisms and etiology remain elusive, previous research has indicated a robust association of SBS5 mutations with aging [26]. Consequently, SBS5, along with SBS1, has been recognized as a paradigmatic example of a mutational signature with clock-like properties. This observation suggests that the mutations specific to the cloned dogs may reflect a ubiquitous genomic aging mechanism, transcending the influences of cellular function or proliferation rates [23].

As a comparison, we also examined the mutational landscape using publicly available sequences of Canine Transmissible Venereal Tumor (CTVT), a parasitic cancer clone that has propagated for thousands of years, which showed a strikingly high frequency of C-to-T substitutions (Figure 2) [27]. This signature, in contrast to the cloned dog-specific somatic mutations, resembles that observed in human melanomas and is associated with UV light exposure [21,22,27], and indicates the heterogeneity of the mutations arising from cloning and tumors.

To investigate whether genes harboring cloned dog-specific somatic mutations show statistically significant enrichment in genes implicated in aging, we employed the comprehensive Human Ageing Genomic Resources (HAGR) database [28]. This database has meticulously curated a list of 954 genes associated with aging through various mechanisms, including cellular senescence and longevity pathways. Among the 420 genes containing clone-specific somatic mutations, 50 (11.9%) were also represented in the HAGR database. Enrichment analysis revealed that the genes with cloned dog-specific mutations are significantly overrepresented in conserved aging pathways (χ^2^ = 21.804, df = 1, *p*-value = 3.02 × 10^−6^).

### 2.3. Molecular Mechanisms Underlying Cloned Dog-Specific Mutations

**Figure 3:**
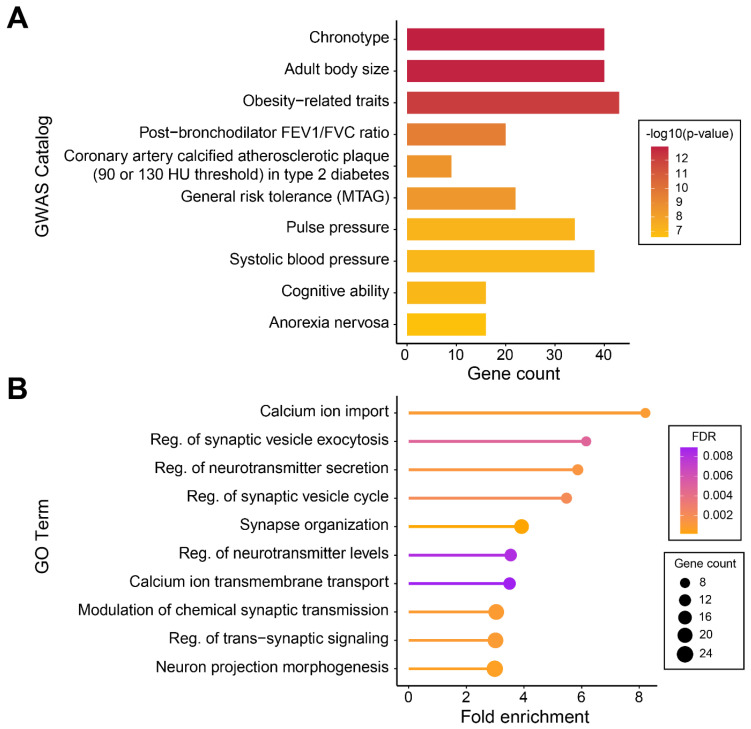
Gene set enrichment analysis of somatic mutations within genes. The top 10 significant results from the GSEA are based on the 420 genes that contain the candidate somatic mutations. (**A**) Chronotype was the most significant GWAS catalog trait. Traits such as adult body size, obesity-related traits, pulse pressure, systolic blood pressure, cognitive ability, and anorexia nervosa are also significant GWAS catalog traits. (**B**) The visualization of the top 10 significant Gene Ontology (GO) terms. The size of each dot represents the number of genes enriched in the term, while the color indicates the level of significance. One of the most enriched processes identified was calcium ion import, with significant enrichment observed in GO terms associated with neurotransmission and synaptic transmission.

To further probe the specific traits and definitive molecular mechanisms associated with cloned dog-specific mutations, we focused on candidate somatic mutations encompassing 420 genes. The enrichment analysis, involving the use of reported human GWAS findings to explore phenotypic alterations due to somatic mutations [29], revealed that the chronotype (*n* = 40, *p*-value = 1.14 × 10^−13^) was the most significant GWAS catalog trait. When the top 10 significant traits were identified, traits including adult body size (*n* = 40, *p*-value = 1.39 × 10^−13^); obesity-related traits (*n* = 43, *p*-value = 6.63 × 10^−13^); post-bronchodilator FEV1/FVC ratio (*n* = 20, *p*-value = 1.86 × 10^−10^); coronary artery calcified atherosclerotic plaque (90 or 130 HU threshold) in type 2 diabetes (*n* = 9, *p*-value = 1.64 × 10^−9^); general risk tolerance (MTAG) (*n* = 22, *p*-value = 2.17 × 10^−9^); pulse pressure (*n* = 34, *p*-value = 3.79 × 10^−8^); systolic blood pressure (*n* = 38, *p*-value = 4.82 × 10^−8^); cognitive ability (*n* = 16, *p*-value = 5.09 × 10^−8^); and anorexia nervosa (*n* = 16, *p*-value = 1.33 × 10^−7^) were also significant GWAS catalog traits (Figure 3, Supplementary Table S3).

As humans age, their sleep timing tends to shift toward an earlier time [30]. Changes in chronotypes are influenced by factors such as hormones and environmental elements [31]. However, genetic variations are believed to account for up to 50% of chronotype variations [32,33,34]. This chronotype undergoes its most substantial fluctuations during early childhood [35,36]. Individuals naturally progress from an early to a late chronotype during youth (15–20 years old), peaking at approximately 20 years of age before shifting to earlier patterns as they age [35,36]. Body size, which was a significant result in the GSEA, also shows rapid growth during the early stages [37,38,39,40]. Large offspring syndrome (LOS) has been reported in cloned animals, suggesting a possible association [2,41]. The occurrence and severity of LOS, one of the most common unexpected events in cloned fetuses, is closely related to embryo culture conditions [42]. LOS is a congenital overgrowth disorder influenced by multi-locus loss of imprinting; global dysregulation of small and long RNAs; and changes in DNA methylation and chromosomal architecture, which affect organ size, cell proliferation, and cell survival [42]. During the early stages, when physical changes manifest rapidly, the body focuses on growth. Therefore, the mutation we identified may impact growth, potentially playing a role in the aging process. Growth and aging are closely related [43,44]. The difference between aging and growth is that aging occurs throughout one’s life, whereas growth occurs in the early stages of life [45,46]. Factors influencing aging can manifest even in childhood, and mutations in genes governing growth-related characteristics may potentially contribute to variations in aging and lifespan [47]. Furthermore, research indicating the inverse relationship between body size and lifespan supports this hypothesis [48]. However, the precise mechanisms underlying these observations remain unclear, and further studies are required.

Obesity can contribute to age-related diseases, including type 2 diabetes and cardiovascular disease [49,50]. Furthermore, obesity can accelerate aging, and age-related declines in energy expenditure and hormonal changes associated with aging may contribute to the development of obesity [51,52]. As aging occurs, not only does the risk of obesity increase, but the incidence of various diseases also increases. Lung function may weaken with aging, and there is a high possibility of developing chronic obstructive pulmonary disease [53,54]. Aging is also a major contributor to the onset of cardiovascular diseases such as coronary artery atherosclerosis [55]. As one ages, arterial stiffness can lead to increased pulse pressure and systolic blood pressure, which may elevate the risk of hypertension and the onset of cardiovascular diseases in the elderly population [56]. Cognitive abilities tend to decline with age, and neurological and biological changes associated with aging can contribute to cognitive impairment [57,58]. Anorexia nervosa is primarily observed in younger age groups but is the most common eating disorder occurring in the elderly population [59,60]. General risk tolerance also appears to be related to age [61]. These findings demonstrate the diverse changes in the body caused by the complex biological interactions associated with aging.

In the Gene Ontology (GO) analysis, a substantial portion of significant terms were related to neurological functions, including calcium ion import (FDR = 9.37 × 10^−4^); regulation of synaptic vesicle exocytosis; regulation of neurotransmitter secretion; regulation of the synaptic vesicle cycle; synapse organization; regulation of neurotransmitter levels; calcium ion transmembrane transport; modulation of chemical synaptic transmission; regulation of trans-synaptic signaling; and neuron projection morphogenesis (FDR < 0.01) (Figure 3B, Supplementary Table S4). Calcium ions are a key regulator of intracellular signaling and serve as a major mediator in triggering the release of neurotransmitters [62]. The calcium ion homeostasis of cells plays a central regulatory role in various neurophysiological aspects, including growth and differentiation, activity potential properties, synaptic plasticity, learning, and memory [63]. Deregulated calcium ion homeostasis can lead to neurodegenerative diseases such as Alzheimer’s disease, Parkinson’s disease, and amyotrophic lateral sclerosis, resulting in neuronal loss [64]. Neurotransmitters also play crucial roles in brain function, behavior, and cognition, serving as key regulators not only in normal aging, but also in neurodegenerative diseases [65]. Dysfunction of the synapses’ functions and structures, where neurotransmitter release and signal transmission occur, is associated with the onset of neurodegenerative diseases [66,67]. Understanding the complex interactions between calcium ions, neurotransmitters, synapses, and synaptic vesicles can help elucidate the causes of age-related neurodegenerative diseases and aid in understanding the cognitive decline and reduction in brain function associated with aging.

### 2.4. Genes with the Highest Somatic Mutation Rates Are Linked to Tumorigenesis

We estimated the mutation rate in order to identify genes with high mutation rates. We annotated the genes where candidate somatic mutations were located and calculated the mutation rate by determining the frequency of mutations per unit gene length. A total of 420 genes were annotated, of which 166 had mutations occurring in two or more positions within the gene (Supplementary Table S5). After sorting according to the mutation rate in descending order, we identified the top 20 genes: *OR51F23*, *L1TD1*, *MRPL21*, *CASP6*, *ZNF777*, *WDCP*, *DLA-79*, *GALC*, *MKI67*, *NIPSNAP2*, *ACTR10*, *CISD2*, *GCM1*, *CKLF*, *FAM98B*, *PRDM4*, *OR6C53*, *TRPV1*, *NUDT6*, and *LAPTM4B* (Table 1, Supplementary Table S6). Of the 20 genes with the highest somatic mutation rates, 9 genes showed associations with tumors.

*L1TD1* (LINE1 type transposase domain containing 1), also known as *ECAT11*, is a gene that encodes a protein involved in early embryogenesis and stem cell pluripotency [68]. The L1TD1 protein is highly expressed in pluripotent cells and interacts with pluripotency regulators OCT4, SOX2, and NANOG [69]. In addition to its role in pluripotency regulation, L1TD1 has been reported to perform important functions in cancer. Expression of L1TD1 has been observed in various cancers such as colorectal carcinoma, ovarian germ cell tumors, and testicular seminomas and non-seminomas [70]. Evidence has been reported suggesting that L1TD1 could be used as a prognostic indicator for colon cancer [71].

*MRPL21* (Mitochondrial Ribosomal Protein L21) is a gene that encodes a protein component of the mitochondrial ribosome. Mitochondrial ribosomal proteins are known to contribute to the regulation of energy metabolism and cell death, and they are closely associated with cancer [72]. In particular, *MRP21* has been demonstrated to be a gene that is selectively expressed in sporadic colorectal cancer biopsies [73]. Furthermore, *MRP21* promotes the growth of hepatocellular carcinoma (HCC), while deficiency of *MRPL21* enhances apoptosis in HCC [74].

*CASP6* (Caspase 6) is a gene that encodes an enzyme belonging to the cysteine-aspartic acid protease (caspase) family. Caspase contributes to the regulation of apoptosis, immune response, and homeostasis [75,76]. Caspase 6 is classified as an apoptotic caspase and is associated with several neurological disorders, such as Alzheimer’s disease and Huntington’s disease [77]. Furthermore, Caspase 6 is also associated with tumors. Although the role of Caspase 6 in the mechanism of cancer pathogenesis is not fully understood, both overexpression and underexpression of caspase have been observed in various tumor tissues [78].

*WDCP* (WD repeat and coiled coil-containing protein), also known as *C2orf44*, has not been extensively characterized regarding its protein function, but it has been investigated in the context of cancer. In colorectal cancer, WDCP has been detected as part of a fusion protein with anaplastic lymphoma kinase (ALK) [79].

*MKI67* (Marker of Proliferation Ki-67) encodes the proliferation marker protein Ki-67. Ki-67 is a biomarker used to estimate the proportion of actively dividing cells for grading tumors [80]. The expression of Ki-67 mRNA is highly correlated with the expression of cell cycle genes and is expressed in all cell cycle phases of vertebrate organisms [81]. However, Ki-67 is not necessarily involved in regulating cell proliferation, and it also plays independent roles in cell proliferation in cancer [81]. Although Ki-67 is not essential for cancer cell proliferation, the expression of Ki-67 influences immune responses, as well as tumor initiation, progression, and metastasis in various cancer types, contributing to tumor growth [82].

*PRDM4* (PR/SET domain 4) encodes the transcription factor of the PR-domain protein family. PR proteins are crucial for cell differentiation and organismal development, but when their regulation is disrupted, they contribute to cancer development [83]. Sequence-tagged site marker and radiation hybrid analyses mapped *PRDM4* to the human chromosome 12q23–q24.1, which is believed to contain tumor suppressor genes associated with ovarian, gastric, and pancreatic cancers [84]. The mapping of *PRDM4* to this specific region suggests that this gene might be involved in the suppression of these cancers.

*TRPV1* (Transient Receptor Potential Cation Channel Subfamily V Member 1) encodes the protein known as the capsaicin receptor [85]. TRPV1 is part of the TRP family of channels, which play a role in sensing nociceptive, thermal, and mechanical sensations [86]. TRPV1 is expressed in both sensory neurons and immune cells [86]. Thus, TRPV1 significantly impacts tumor growth and metastasis by modulating neurogenic inflammation and immune responses [87].

*NUDT6* (Nudix hydrolase 6) is known as *GFG1* and is an antisense gene of *FGF-2* (fibroblast growth factor-2). Proteins of this gene may contribute to the expression of *FGF-2* and the regulation of cell proliferation [88,89]. Due to the association between the expression of the Nudix hydroxylase family of genes and cancer, it is believed that NUDT6 may also contribute to tumorigenesis and the progression of tumors [90,91,92].

*LAPTM4B* (Lysosomal Protein Transmembrane 4 Beta) is considered an oncogene. The proteins of *LAPTM4B* promote cell growth and proliferation in various cancers such as HCC, gallbladder carcinoma, and non-small cell lung cancer [93]. In particular, LAPTM4B-35 is upregulated in various cancers including HCC, breast cancer, gastric cancer, lung cancer, colon cancer, gallbladder cancer, extrahepatic cholangiocarcinoma, and ovarian carcinoma [94]. Furthermore, *LAPTM4B* promotes tumor metastasis and inhibits apoptosis [93].

Overall, some of the genes we have identified contribute to tumorigenesis and suppression, cell proliferation, cell death, and genetic regulation. Aging and cancer are closely linked, with numerous shared mechanisms [95]. One of the important drivers of aging and cancer is DNA damage [96]. Most DNA damage can be corrected through the DNA repair process, but if DNA damage is not repaired due to errors in this process, it can lead to an accumulation of somatic mutations, which in turn may disrupt normal cell function, promote cellular senescence, or lead to the development of cancer.

### 2.5. Limitations of the Study

The main obstacle in researching somatic mutations in cloned animals is the negligible genetic differences between the original and its clones, which are fewer than those found between identical twins. Additionally, our study navigated the somatic mutations identified from the comparison between one original dog and two cloned dogs derived from that original donor. This underscores the need to distinguish whether genetic disparities stem from genuine somatic mutations or systemic sequencing errors—a determination that can be validated through additional and independent pairs of original and cloned dogs. However, given that few studies have investigated somatic mutations in cloned animals, and no cases have been reported regarding the biological functions of these somatic mutations, our study provides an essential foundation for future studies to examine the molecular mechanisms underlying cloning in the context of genome stability.

## 3. Conclusions

We compared variants from an original dog with those in the cloned dogs and identified 6168 somatic mutations unique to the cloned dogs. We demonstrated that these mutations were significantly associated with traits related to human aging, and we showed that the pattern of the mutational signature mirrored previously reported signatures distinctive to aging, particularly during early life stages. Regarding mutation frequency, the genes were strongly associated with tumors, cellular apoptosis, and proliferation. Overall, our study highlights the substantial genomic variability in clones, and the somatic mutations identified in this study could provide mechanistic insight into genomic stability.

## 4. Materials and Methods

### 4.1. Sample Data

In another study, cloned dogs were produced via SCNT using ear fibroblasts from a 5-year-old male German Shepherd [17]. Whole genome sequences of the original dog were generated from the whole blood of a 6-year-old German Shepherd. Whole genome sequences of the cloned dogs were generated from the whole blood of two 4-month-old cloned dogs with normal phenotypes (NT2, NT4). Genomic DNA was extracted from the blood of the two cloned dogs and the original dog with a Wizard Genomic DNA Purification Kit (Promega, Madison, WI, USA). DNA libraries for each sample were prepared using the TruSeq Nano DNA Sample Prep Kit (Illumina, San Diego, CA, USA). Whole genome sequencing was performed using the Illumina HiSeq 2500 sequencing platform (Illumina, San Diego, CA, USA). Sequence reads were mapped to the CanFam 3.1 reference genome using the bowtie2 algorithm (v2.3.5.1), sorted with Samtools (v1.9) [97,98,99]. Detailed information and coverage rates for the original and cloned dogs are provided in Supplementary Table S1. The newly sequenced dog genomes in this study are publicly available from the European Molecular Biology Laboratory’s European Bioinformatics Institute (accession no. E-MTAB-13924).

### 4.2. Candidate Somatic Mutation Identification

To identify somatic mutations, we used Mutect2 in GATK [18]. We aimed to identify somatic mutations in NT2 and NT4 compared to the original dog. In NT2 and NT4, 42,184 and 38,821 heterozygous variants were identified, respectively. Variants with a read depth of 50 or less, or those found in only one cloned dog, were filtered out. Eventually, 6168 variants were categorized as candidate somatic mutations. The functional annotation of the variants was performed using SnpEff software (v4.3 t) [19]. Next, 38 variants with a moderate-to-high impact on SnpEff annotation were selected. We then leveraged the publicly available sequences of 196 domestic dogs to verify the presence/absence of each candidate somatic variation from the list of 19,310,643 germline variants found in the general population [20] (Supplementary Table S7), after applying the missing rate threshold of 10%. The cloned dog-specific allele frequency was estimated and is provided in Supplementary Table S2.

We performed gene annotation for the cloned dog genome using the Ensembl gene annotation information for the CanFam3.1 reference genome assembly and the variant positions obtained from the whole genome sequencing of cloned dogs, annotating a total of 15,096 genes. Among the 15,096 annotated genes in the cloned dog genome, 420 genes were annotated with somatic mutations.

### 4.3. Gene Set Enrichment Analysis

GSEA was conducted using FUMA GWAS. GENE2FUNC of FUMA GWAS was used to identify the gene set reported in the GWAS catalog [29]. The GTEx v.8 dataset was used for gene expression analysis. When conducting FUMA GWAS, the background gene type was set as protein coding, and we applied the Benjamini–Hochberg method (FDR) for multiple test correction during gene set enrichment testing. We set the threshold for the maximum adjusted *p* value for gene set association at less than 0.01 and required a minimum overlap of at least two genes with gene sets (Supplementary Table S3). Bar plots were constructed using the R package ggplot2.

Gene Ontology (GO) analysis was performed using ShinyGO v.0.80 and StringDB software (v11.5) [100]. For statistical significance, the 41 significant biological processes (BPs), 45 cellular components (CCs), and 21 molecular functions (MFs) were identified based on FDR < 0.01. These were sorted by fold enrichment (Supplementary Table S4). The plots for BPs were constructed using the R package ggplot2.

### 4.4. Mutation Rate

During the gene annotation process, 1190 genes were annotated using candidate somatic mutations, of which 420 genes contained candidate somatic mutations (Supplementary Table S5); of these genes, only those with mutations occurring at least twice were selected, resulting in 166 genes. For these 166 genes, the mutation rate was calculated by dividing the number of mutations in each gene by its length.

### 4.5. Aging-Related Gene Database

To identify aging-related genes, we employed the HAGR (Human Ageing Genomic Resources) database, which contains a curated list of genes associated with aging in humans. From this database, we filtered genes that have been validated as being associated with aging processes in humans. Specifically, we compiled a list of 1170 unique genes by combining 307 genes annotated as aging-related in humans; 611 genes from human microarray studies on aging; and 358 significant genes from the LongevityMap resource, after excluding any overlapping or duplicate gene entries. By cross-referencing the list of 1170 aging-related genes from HAGR with the 15,096 annotated genes in the cloned dogs, we identified 954 genes that were present in both datasets, which were designated as aging-related genes. The remaining genes not included in the HAGR-derived list were considered not associated with aging. Out of the 420 genes containing somatic mutations, 50 genes overlapped with the 954 aging-related genes. The statistical significance of this overlap was evaluated using the chi-squared test, implemented in the R statistical software (v4.3.3) environment. Contingency tables (2 × 2), along with tests of independence and measures of association, were performed with R software, using the chi-squared test with Yate’s continuity correction.

## Figures and Tables

**Table 1:** The top 20 genes with the highest mutation rates. Upon identifying 420 genes with candidate somatic mutations, we determined the mutation frequency for each gene by dividing the mutation count by the respective gene’s length. When ranked by frequency, the 20 genes with the most pronounced mutation frequencies encompassed functions, including olfactory reception, tumorigenesis, apoptosis, and cell proliferation.

Gene	Chr	Length	Count	Frequency
*OR51F23*	2	930	3	3.23 × 10^−3^
*L1TD1*	5	5205	2	3.84 × 10^−4^
*MRPL21*	1	13,363	4	2.99 × 10^−4^
*CASP6*	3	13,647	4	2.93 × 10^−4^
*ZNF777*	1	22,897	6	2.62 × 10^−4^
*WDCP*	1	19,464	5	2.57 × 10^−4^
*DLA-79*	1	27,794	6	2.16 × 10^−4^
*GALC*	8	57,150	9	1.57 × 10^−4^
*MKI67*	2	26,464	4	1.51 × 10^−4^
*NIPSNAP2*	6	33,134	5	1.51 × 10^−4^
*ACTR10*	8	31,824	4	1.26 × 10^−4^
*CISD2*	3	16,071	2	1.24 × 10^−4^
*GCM1*	1	16,477	2	1.21 × 10^−4^
*CKLF*	5	16,928	2	1.18 × 10^−4^
*FAM98B*	3	34,924	4	1.15 × 10^−4^
*PRDM4*	1	26,340	3	1.14 × 10^−4^
*OR6C53*	3	17,700	2	1.13 × 10^−4^
*TRPV1*	9	26,955	3	1.11 × 10^−4^
*NUDT6*	1	31,071	3	9.66 × 10^−5^
*LAPTM4B*	1	86,062	8	9.30 × 10^−5^

## Data Availability

The data presented in the study are openly available in E-MTAB-13924.

## Supplementary Materials

The following supporting information can be downloaded at: https://www.mdpi.com/article/10.3390/genes15060801/s1, Table S1: Sample information; Table S2: Moderate and High effect mutation information; Table S3: Result of the GWAS catalog; Table S4: Result of the Gene Ontology; Table S5: Mutation Rate; Table S6: Gene information; Table S7: 196 dog population information. References [101,102] are cited in the supplementary materials.
